# Identification of the Raman Salivary Fingerprint of Parkinson’s Disease Through the Spectroscopic– Computational Combinatory Approach

**DOI:** 10.3389/fnins.2021.704963

**Published:** 2021-10-26

**Authors:** Cristiano Carlomagno, Dario Bertazioli, Alice Gualerzi, Silvia Picciolini, Michele Andrico, Francesca Rodà, Mario Meloni, Paolo Innocente Banfi, Federico Verde, Nicola Ticozzi, Vincenzo Silani, Enza Messina, Marzia Bedoni

**Affiliations:** ^1^IRCCS Fondazione Don Carlo Gnocchi ONLUS, Milan, Italy; ^2^Università degli Studi di Milano-Bicocca, Milan, Italy; ^3^Laboratory of Neuroscience, Department of Neurology-Stroke Un, IRCCS Istituto Auxologico Italiano, Milan, Italy; ^4^Department of Pathophysiology and Transplantation, “Dino Ferrari” Center, Università degli Studi di Milano, Milan, Italy; ^5^Aldo Ravelli Center for Neurotechnology and Experimental Brain Therapeutics, Università degli Studi di Milano, Milan, Italy

**Keywords:** Parkinson’s disease, saliva, Raman spectroscopy, classification model, deep learning

## Abstract

Despite the wide range of proposed biomarkers for Parkinson’s disease (PD), there are no specific molecules or signals able to early and uniquely identify the pathology onset, progression and stratification. Saliva is a complex biofluid, containing a wide range of biological molecules shared with blood and cerebrospinal fluid. By means of an optimized Raman spectroscopy procedure, the salivary Raman signature of PD can be characterized and used to create a classification model. Raman analysis was applied to collect the global signal from the saliva of 23 PD patients and related pathological and healthy controls. The acquired spectra were computed using machine and deep learning approaches. The Raman database was used to create a classification model able to discriminate each spectrum to the correct belonging group, with accuracy, specificity, and sensitivity of more than 97% for the single spectra attribution. Similarly, each patient was correctly assigned with discriminatory power of more than 90%. Moreover, the extracted data were significantly correlated with clinical data used nowadays for the PD diagnosis and monitoring. The preliminary data reported highlight the potentialities of the proposed methodology that, once validated in larger cohorts and with multi-centered studies, could represent an innovative minimally invasive and accurate procedure to determine the PD onset, progression and to monitor therapies and rehabilitation efficacy.

## Graphical Abstract

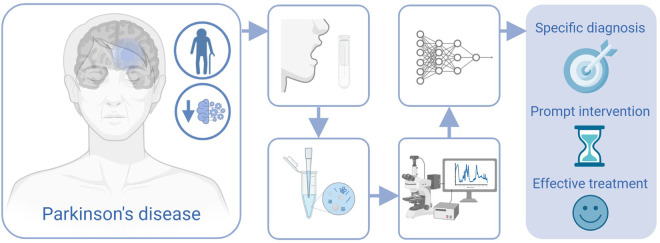

## Introduction

Parkinson’s disease (PD) is one of the most common neurodegenerative disorders occurring in the elderly, associated with the inactivation of dopaminergic neurons in the substantia nigra and with the appearance of Lewy bodies made of abnormal α-synuclein (Boller et al., 1980; Davie, 2008). Epidemiologically, PD is the second most relevant neurodegenerative disorder after Alzheimer’s disease (AD), with an increasing burden in aging society (Berg, 2008). The PD diagnosis mainly relies on clinical motor symptoms, which occur generally decades after the pathology onset (Sveinbjornsdottir, 2016; Lindestam Arlehamn et al., 2020). This lag time hampers the detection of the earliest phases of the disease and the time at which the treatment with neuroprotective drugs could have the greatest effect (Berg, 2008). The further onset of cognitive impairment of variable degrees, common in various neurodegenerative disorders, before and during PD progression, lead to the worsening of the clinical diagnosis and prolonging the diagnostic period for a clear PD identification (Aarsland et al., 2010; Martinez-Horta and Kulisevsky, 2019). In this frame, researchers are focused on the identification of a measurable and easily collectable PD biomarker able to identify the disease onset also in the preliminary pathological phases [levels 1 and 2 of Braak’s staging (Braak et al., 2003)], to monitor the therapeutic and motor-neuronal rehabilitation efficacies and also to clearly distinguish the different forms of parkinsonism (Gelb et al., 1999). Up to now, most of the potential biomarkers have been identified in the cerebrospinal fluid (CSF) and in peripheral blood (serum and plasma) (Parnetti et al., 2019). α-Synuclein, and the autosomal enzymes involved in synuclein degradation, are actually the most promising biomarkers and have been widely studied in CSF and blood, playing a central role in PD and other synuclein aggregation disorders (Gao et al., 2015; Eusebi et al., 2017; Parnetti et al., 2017, 2019; Poewe et al., 2017). Other molecules involved directly in PD onset or in the organism response to the disease have been characterized and proposed as potential biomarkers, including amyloid species, microRNA, specific cytokines expression patterns and molecules associated to the damages of radical oxygen species (ROS) (Zhang et al., 2008; Devic et al., 2011; Bäckström et al., 2015; Liang et al., 2015; Lleó et al., 2015; Kori et al., 2016; Bougea et al., 2019; Maass et al., 2019; Parnetti et al., 2019; Sancho Cantus et al., 2019). The main limitations related to all the described molecules concern the invasiveness of the sample collection procedure (i.e., CSF), the sharing of several biomarkers with other similar pathologies (PD and AD) and to the techniques used for their characterization (Berg, 2008). The application of methodologies such as ELISA, blotting assays or mass spectroscopy focalize the investigation on one, or few, targets for each analysis with long preprocessing steps and high-cost procedures and materials (McKhann et al., 2011; Postuma et al., 2015; Parnetti et al., 2019). These limitations make difficult the characterization of the whole biomarkers presence inside the chosen biofluid, hiding the complex expression patterns that can precisely identify the onset of a neurological disorder, such as the entire proteome involved in the inflammatory response or the biological product of the ROS pathway (Berg, 2008; Reed, 2011; Maciejczyk et al., 2020). Therefore, the identification of a fast methodology able to determine a global biomarker with a minimally invasive procedure is of crucial importance for the PD diagnosis and for the monitoring of therapeutic and rehabilitative efficacy. Saliva is a complex biofluid collectable with a minimally invasive procedure, containing several biological molecules (i.e., protein, enzymes, lipids, nucleic acids, carbohydrates, metabolites, and hormones) shared with blood or CSF due to the physiological transport processes (Huang, 2004; In et al., 2009). Among these molecules, different studies reported the presence of potential biomarkers directly correlated with PD onset, including heme-oxygenase-1 and cysteine protease DJ-1 (Kang et al., 2014; Bäckström et al., 2015; Song et al., 2018; Bougea et al., 2019). Therefore, the characterization of the entire pattern of biomolecules contained in saliva of PD patients could be of crucial importance in order to assess differences in composition and attributing at the same time the origins of these differences. To overcome the detection and velocity limits of the methodologies used nowadays, one of the most promising approaches regards Raman spectroscopy (RS) that is able to detect the concomitant presence, concentration, mutation, environment, and interactions of different biological species inside a target biofluid (Devitt et al., 2018). The output of the RS analysis consists in a complex spectrum containing the complete and sensitive (10^–8^ to 10^–15^M) biochemical information in the so called “Raman fingerprint” (Feng et al., 2015). RS has already been proposed for the diagnosis of neurodegenerative diseases (Gualerzi et al., 2019; Carlomagno et al., 2020b) and the RS analysis of saliva has been applied for forensic purposes, chemotherapy monitoring, drug abuse evaluation, and for the diagnosis of different pathologies including tumors, viral infections, amyotrophic lateral sclerosis, AD, and for the detection of salivary α-synuclein in PD patients (Farquharson et al., 2005; Virkler and Lednev, 2010; Gonchukov et al., 2012; Li et al., 2012; Andreou et al., 2013; Al-Nimer et al., 2014; Radzol et al., 2014; Feng et al., 2015; Qiu et al., 2016; Ralbovsky et al., 2019; Carlomagno et al., 2020a). The aim of the present work regards the application of the RS for the analysis of saliva collected from PD patients and compared with related healthy subjects and other pathological controls to verify statistical differences able to create an automatic classification model. The methodology involves the modification of a previous RS protocol for the analysis of saliva (Carlomagno et al., 2020a), in which the time for sample preparation has been reduced, whereas the total amount of biological molecules that can be monitored has increased. The collected data were used for the characterization of the PD Raman fingerprint by means of the multivariate analysis (MVA), creating a classification model able to discriminate the membership of the single spectra. In order to enhance the discriminative power from the single-spectra level to a patient-level enabling the deployment of the predictive framework for diagnostic purposes, more complex non-linear interactions need to be represented. For this reason, machine learning (ML) and deep learning (DL) models have been investigated, further automating the data analysis for unveiling hidden patterns that correlate with the pathology. In particular, DL has emerged as one of the most focused research aimed at learning features and directly building predictive models from large-scale raw datasets (Chatzidakis and Botton, 2019), with excellent performances in many biochemical fields including spectroscopy (Alakwaa et al., 2018), metabolomics (Eraslan et al., 2019), and genomics (Gautam et al., 2015). In fact, DL based methods are well adapted to highlight the complex connections within high dimensional data provided by RS (Gautam et al., 2015; Lussier et al., 2020). In this work, the Raman data collected from PD patients, AD and mild cognitive impairment (MCI) patients and healthy controls (CTRL) were processed creating a MVA-based classification model able to individuate the single Raman fingerprint of PD patients with sensitivity, specificity, and accuracy of respectively, 97, 98, and 98% for the single-spectrum classification model. The consecutive application of convolutional neural network (CNN), combined with a data augmentation strategy to enrich the training dataset and a sequential model-based optimization (SMBO) for the computation of the hyper-parameter configuration (Hutter et al., 2011; Feurer and Hutter, 2019), revealed sensitivity, specificity, and accuracy of 90, 94, and 89% in the identification of PD patients between the considered experimental groups. The extracted parameters from the Raman database correlated with the clinical scores used for the diagnosis and monitoring of PD progression.

## Materials and Methods

### Materials

All the materials were purchased from Sigma-Aldrich (United States) and used as received if not differently specified. Salivette^®^ swabs for the saliva collection were purchased from Sarstedt (Germany, catalog number S1 1534). Mini Bin aluminum foils (Sigma-Aldrich, United States, catalog number Z691569-1EA) were used as Raman substrate. All the materials were used following the manufacturer’s instructions without further purification steps. All the described procedures were performed in accordance with relevant guidelines, regulations, and ethical standards. The procedures were approved by the Ethical Committee of the institution in which the experiments were done or in accord with the Helsinki Declaration of 1975.

### Patients Selection

All the recruited participants for the exploratory study provided written informed consent and the study was approved by the institutional review board at IRCCS Fondazione Don Carlo Gnocchi ONLUS on March 12th, 2018. The study was not pre-registered and no randomization and no blind methods were applied. PD patients and CTRL recruitment took place at the Neurology Unit of IRCCS Fondazione Don Carlo Gnocchi ONLUS in Milan (Italy), between June 2019 and June 2020. AD participants were recruited in the Department of Neurology-Stroke Unit of IRCCS Istituto Auxologico Italiano in Milan (Italy). PD patients were diagnosed according to the Movement Disorder Society Clinical Diagnostic Criteria for PD (Postuma et al., 2015), excluding patients affected by vascular parkinsonism (with cerebrovascular disease, as indicated by brain imaging computed tomography or magnetic resonance imaging, or by the presence of symptoms that are consistent with stroke). Moreover, brain tumor, drug-induced parkinsonism, other known or suspected causes of parkinsonism (e.g., metabolic, etc.), or any suggestive features of a diagnosis of atypical parkinsonism, severe speech problems and poor general health, concomitant neurologic, and/or psychiatric diseases were also excluded. The Hoehn and Yahr (H&Y) and the Movement Disorder Society – Unified Parkinson’s Disease Rating Scale motor part III (UPDRS III) criteria were adopted for the evaluation of the disease stage and the symptoms severity.

Levodopa equivalent daily doses (LEDD) prescribed at the time of saliva collection were registered for each patient. Patients with AD patients and MCI due to AD were diagnosed according to the clinical criteria described by Albert et al. (2011) and McKhann et al. (2011), excluding individuals with neurological or major psychiatric comorbidities. CTRL were matched for age and gender to the AD and PD patients in order to limit sex hormone variability in saliva that can affect the Raman signature (Muro et al., 2016). Exclusion criteria for CTRL, PD, and AD were a continuous drug administration (e.g., anti-hypertensive) and the presence of chronic or/and inflammatory oral or systemic diseases or infections. For this study, a total number of 23 PD (*n* = 23), 10 AD (*n* = 10), and 33 CTRL (*n* = 33) were selected for the saliva collection. The number of PD patients was enrolled in the study without a preliminary sample size calculation. Statistical comparison between the groups was performed using the ANOVA two-tailed *t*-test and Chi-square test. For all the participants, demographic, personal, and clinical data were recorded. All the demographic and clinical information regarding the subjects involved in the study are reported in Table 1.

**TABLE 1:** Number, age, male sex percentages, Hoehn and Yahr (H&Y), Unified Parkinson’s Disease Rating Scale motor part III (UPDRS III), levodopa equivalent daily doses (LEDD) of subjects affected by Parkinson’s disease (PD), by Alzheimer’s disease (AD), and healthy subjects (CTRL).

	**PD**	**AD**	**CTRL**
Number	23	10	33
Age (*p* = 0.246)	69.9 ± 8.8	78.6 ± 7.9	63.5 ± 8
Male sex (*p* = 0.14)	74%	80%	66.6%
H&Y	2.19 ± 0.7	–	–
UPDRS III	31.7 ± 14.5	–	–
LEDD	466 ± 220	–	–

*Subject’s age in different groups was compared through ANOVA two-tailed *t*-test. Sex percentage was tested through Chi-square test. Values are followed by standard deviations (±*n*) or by the relative percentage (*n*%).*

### Saliva Collection and Raman Analysis

The saliva collection procedure was performed following the instructions reported for Salivette^®^. Briefly, the swab was placed in the mouth of the subject and chewed for 1 min in order to stimulate salivation. To limit the results variability, the salivary collection time was fixed at an appropriate period from the last meal (2 h) and from teeth brushing (2 h) in the morning, keeping the same collection time for all the participants. Storage time and temperature (4°C), time between the collection and Raman analysis, smoking and dietary habits, oral and respiratory infections, gingivitis or periodontitis, and recent dental surgeries were recorded. The swab was then centrifuged at 1,000 *g* for 2 min in order to recover saliva. The Raman acquisition procedure and part of the data processing were adapted from previously published works (Carlomagno et al., 2021a,b). Raman spectra were acquired using the Raman microscope Aramis (Horiba Jobin-Yvon, France), equipped with a laser source at 785 nm at 512 mW power emission. The analysis was performed after instrument calibration with the reference band of silicon at 520.7 cm^–1^, using 30 s acquisition time. A drop (3 μL) of saliva was casted on an aluminum foil and dried at room temperature in order to achieve the surface enhanced Raman spectroscopy (SERS) effect (Muro et al., 2016; Carlomagno et al., 2020a). Raman analysis was performed using a square-map (80 μm × 60 μm) close to the center of the drop, with the acquisition of at least 30 points for each subject. The acquisition range was set between 400 and 1600 cm^–1^. All the analyses were performed using a 50× objective (Olympus, Japan) and with a spectral resolution of 0.8 cm^–1^/step. The laser grating was set at 600 while the hole was kept at 400.

### Data Processing, Statistical Analysis, and Single Spectrum Classification Model

For successfully applying both MVA and ML, the spectral preprocessing step is crucial as it can strongly affect the classification performances. The raw acquired spectra were fitted with a fifth-degree polynomial baseline and normalized (unit vector) using the incorporated acquisition software LabSpec 6 (Horiba, France). With the same software, all the data were despiked and resized, aligning the spectra to the peak at 1001 cm^–1^. The contribution of the aluminum substrate was subtracted from each spectrum. For spectral representation, the second-degree Savitzky–Golay smoothing method was applied. Artifact spectra produced due to high fluorescence (saturation) or no signal (laser *Z*-axis shift) were identified and removed using the incorporated software LabSpec 6 (Horiba, France). At least 20 spectra for each subject were maintained for further statistical analysis. The MVA analysis was performed using principal component analysis (PCA) and linear discriminant analysis (LDA) on the three experimental groups, reducing the dimension of the data and highlighting the most relevant trends. The first 15 Principal Components (PCs) were used to create the LDA-based classification model to discriminate the data maximizing the variance between the groups, and to avoid data overfitting analyzing the cumulative loading of PCs of 78.3%. The error rate, accuracy, sensitivity, and specificity in spectra attribution of the model were tested using leave-one-out cross-validation (LOOCV) and confusion matrix. The receiver operating characteristic (ROC) curve was calculated using the MVA results on the classification model (Gualerzi et al., 2019). The Matthews correlation coefficient (MCC) was calculated to assess the quality of the binary classification (PD versus no PD). ANOVA test and Chi-square test were applied to verify the statistical relevant differences between the experimental groups. The correlation between the MVA results and clinical and paraclinical tests or scores was calculated using Pearson’s correlation, while the partial correlation coefficient was used to assess the effect of the covariates (age and sex) on the final scores. Correlation results were considered statistically relevant for *p*-values < 0.05. This section of the statistical analysis was performed using OriginPro 2018 (OriginLab, United States).

### Machine Learning Data Preparation

The applied pipeline included four different phases: data preprocessing and augmentation, model selection, model tuning, and model evaluation. In the preprocessing steps, we performed the removal of the outlier not-informative spectra (artifacts) and the realignment of the Raman shift axis, by means of a linear interpolation (Díez-Pastor et al., 2020), resampling each spectrum on a given grid of 900 points between 400 and 1600 cm^–1^. The signal given by the intensity of the aluminum substrate was subtracted from the original signal and the background noise given by the fluorescence of the samples, was removed by baseline fitting with a sixth-degree polynomial.

Despiking was performed by means of Whitaker–Hayes algorithm (Whitaker and Hayes, 2018) allowing the removal of any spike given by cosmic rays (threshold: 3.5; size of the neighborhood: 11). Finally, we tested Standard Normal Variate, Max value, Min–Max, and L2 techniques for intensity normalization. The results obtained showed substantial robustness of the classification models with respect to these normalization methods. Data augmentation was performed generating new synthetic data samples by applying variations and distortions to the original data (injection of Gaussian noise, offset in Raman shift and slope) to maximally exploiting their intrinsic invariances and partially overcoming data scarcity (Shorten and Khoshgoftaar, 2019). Since in our data set the PD–AD classes appear to be unbalanced in favor of the CTRL class, new patients’ data have been generated according to a different multiplication factor based on the degree of imbalance.

### Application of Learning Models for the Patient Classification Model

Support vector machine (SVM) and random forest (RF) were implemented as baseline ML algorithms and compared with different DL models, namely fully connected neural networks (FCNN) and 1D CNNs. A grid search approach was applied to find suitable hyper-parameters configurations in ML models, while Tree Parzen Estimator (TPE) sampling and SMBO has been used to optimize our FCNN and CNN, respectively. The DL-related hyper-parameters optimization task was performed through the Optuna framework (Akiba et al., 2019) in combination with Scikit-Optimize Library (Head et al., 2018). This Bayesian optimization framework allows the integration, in addition to the native TPE sampler, an SMBO module provided by Scikit-Optimize. The hyper-parameters optimization involved two main phases: the overall DL model structure optimization (selecting the number of convolutional or dense layers) and the tuning of the fine hyper-parameters. SMBOs were applied with Gaussian processes and RF regressions as base estimators along the process, both in combination with specific acquisition function. All the optimizations were performed in 10-folds cross-validation and the objective function was minimized on the average classification error. The models performances were evaluated on their ability in discriminating between PD, AD, and CTRL subjects according to the majority label assigned to their spectra. To control overfitting, we regularized our DL model by the intensive use of dropout masks in the fully connected layers responsible for the classification. In addition, we applied early stopping to avoid overtraining that could potentially harm generalization in our settings. Furthermore, to overcome any classification bias given by an arbitrary test-set choice, we applied the leave-one-patient-out cross-validation (LOPOCV), a robust and stable procedure where each test-fold is composed by the entire set of spectra from a single patient that guarantees a most accurate estimation of the model performances. The entire pipeline was coded in Python, and while ML algorithms have been implemented through the Scikit-Learn Library (Pedregosa et al., 2011), for the DL ones we exploited Keras, the Tensorflow high-level API (GitHubkeras-team/keras, 2021).

## Results

### Raman Analysis of Saliva

**FIGURE 1:**
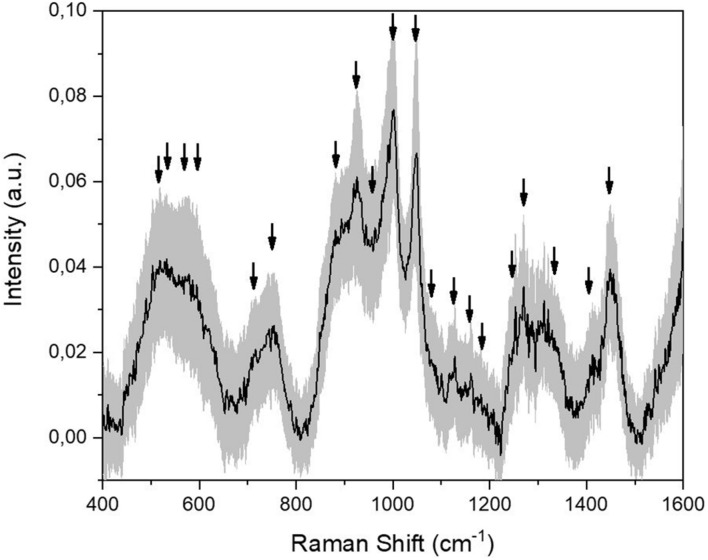
Average Raman signal obtained from the collected CTRL salivary samples (*n* = 33). Black arrows indicate the identified peaks. The gray band represents the standard deviation.

The modification of a previous optimized protocol was adopted for the analysis of saliva collected from 23 PD patients, 10 AD patients, and 33 CTRL (Carlomagno et al., 2020a). The principal modification regarded the removal of sample filtration step with 3-kDa filters, resulting in a wider range of molecules detected and in a faster analytical procedure. The average spectra obtained from all the collected CTRL samples (*n* = 33) is presented in Figure 1. The detailed signal provides an overview on the species that mostly contribute to the Raman spectrum, with attributed peaks at 517, 532, 578, 619, 715, 750, 870, 920, 978, 1001, 1047, 1077, 1102, 1125, 1203, 1244, 1268, 1346, 1415, and 1444 cm^–1^ (Figure 1, black arrows). The highlighted peaks and bands regard the typical Raman signal provided by salivary samples analyzed using aluminum substrates (Table 2; Muro et al., 2016). The most important signal attribution regards the peak at 750 cm^–1^ related to the O–O stretching vibration in oxygenated proteins. The peaks at 870 and 1125 cm^–1^ can be attributed to the C–N stretching and to the CH_3_ rocking in protein backbone, respectively, with the peak at 1001 cm^–1^ related to the ring breathing of aromatic amino acids and the signal at 1444 cm^–1^ assigned to the C–H stretching of glycoproteins, mostly obtained from mucins (Movasaghi et al., 2007; Gonchukov et al., 2012). The peaks at 1002 and 1346 cm^–1^ regard, respectively, the secondary bands of Amide I and the principal band of the Amide III, which are directly correlated with the secondary structures of the protein contained in saliva (Rygula et al., 2013). The associated standard deviation reveals high variability among the tested samples, probably due to the different physiological and pathological states of the subjects involved in the study (Figure 1, gray band). The great part of attributed modes regards the vibration of molecules belonging to the protein, lipid, nucleic acid, and saccharide families, providing a global overview on the distribution of the species inside the biofluid (Table 2).

**TABLE 2:** Attribution of the most prominent peaks obtained from Raman salivary analysis (±8 cm^–1^), based on reported literature (Movasaghi et al., 2007; Gonchukov et al., 2012; Muro et al., 2016).

**Raman shift (cm**^–1^)	**Attribution**
517	Phosphatidylinositol
532	Ester of cholesterol
578	Tryptophan, cytosine, guanine
619	Phenylalanine twisting
715	Phospholipids
750	O–O stretching in protein
870	C–N stretching in protein, tyrosine ring breathing
920	Glucose/glycogen
978	C–C stretching of phosphorylated proteins
1001	Aromatic ring breathing
1047	C–C in plane bending
1077	C–C bond of lipids
1102	Secondary bands of Amide I
1125	Tyrosine, phenylalanine, CH_3_ rocking in protein
1203	Tyrosine
1244	Nucleic acids/secondary bands of Amide III
1268	C–H bond of phospholipids
1346	Amide III
1415	C–H deformation
1444	C–H stretching of glycoproteins

**FIGURE 2:**
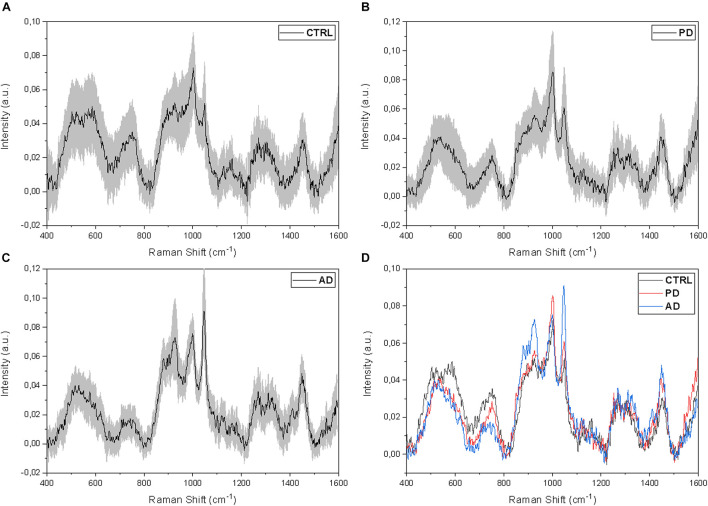
Average salivary Raman spectra of the **(A)** CTRL (*n* = 33), **(B)** PD (*n* = 23), and **(C)** AD (*n* = 10) experimental groups. The gray bands represent the associated standard deviations. **(D)** Overlapped average Raman spectra of the three analyzed groups.

**FIGURE 3:**
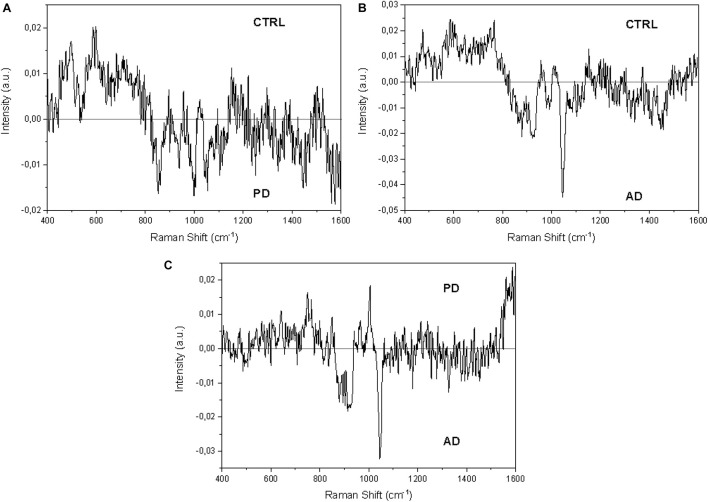
Subtraction Raman spectra of **(A)** the average CTRL versus the average PD signals, **(B)** the average CTRL versus the average AD signals, and **(C)** the average PD versus the average AD signals.

The proposed procedure allows the collection of detailed spectra using a very fast protocol that foresees a minimal sample preparation. The molecular content, as well as the information provided by the spectra, are detailed and repeatable giving an optimal point of view on the biochemical species present in saliva. For these reasons, we analyzed 33 CTRL, 23 PD, and 10 AD saliva samples following the procedure described previously. Figure 2 shows the Raman signals collected from the saliva of CTRL (*n* = 33; Figure 2A), PD (*n* = 33; Figure 2B), and AD (*n* = 10; Figure 2C) groups. Considering the intra-group variability, the preliminary analysis highlights a higher standard deviation value of the CTRL group (Figure 2A) respect to the pathological groups (Figures 2B,C), indicating a potential specific biochemical equilibrium during the pathological onset. As it is possible to notice from the overlapped average Raman spectra, the main differences between the groups can be attributed only to variations in intensities and presence of specific peaks, with negligible signal shifts. The remarked differences in peak intensities are probably due to variations in the molecular concentration and distribution between the physiological and pathological states (Figure 2D). In order to highlight the principal spectral discrepancies between the experimental groups and to investigate potential new discriminatory regions, subtraction spectra were obtained by comparing the signal intensities of CTRL, PD, and AD at different wavelengths (Figure 3). All the differences in intensity (ΔI) were considered for values of ΔI ≥ 0.01. The main differences between CTRL and PD (Figure 3A) are due to the peaks at 496, 595, 678, 715, 770, 829, 850, 939, 1001, 1047, 1102, 1244, 1346, 1415, 1444, and 1571 cm^−1^. Similarly, the main differences between CTRL and AD were identified at 472, 593, 641, 750, 770, 870, 920, 1047, 1372, 1415, and 1444 cm^–1^ (Figure 3B), while regarding the comparison between PD and AD peaks at 643, 750, 767, 870, 920, 1001, 1047, 1181, and 1326 cm^–1^ showed the main differences (Figure 3C). These differences compared with the already attributed peaks (Table 2) are mainly due to the potential modifications of proteins, lipids, and carbohydrates and confirm previous observations on the overlapped spectra (Figure 2D). In particular, in the subtraction spectrum between PD and AD patients (Figure 3C), the most prominent peaks in PD were related to protein (643, 750, 1001, and 1580 cm^–1^ are peaks related to single amino acids while the peak at 1540 cm^–1^ regards the Amide II band) and to phosphatidylinositol (770 cm^–1^). The same difference was encountered between PD and CTRL (Figure 3A), but in this case the resultant intensity with the relative error propagation (ΔI ≤ 0.01) was not considered statistically significant.

### Classification Models

#### Single Spectra Classification Model

**FIGURE 4:**
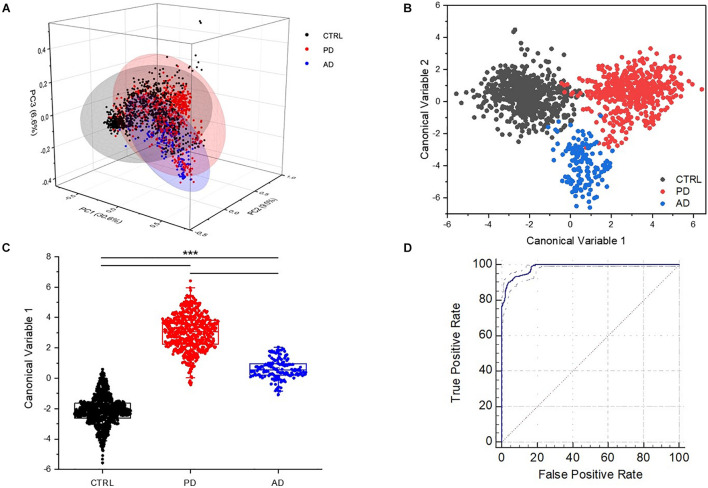
**(A)** Principal component analysis (PCA) in three axis distribution (*X* = PC1; *Z* = PC2; *Y* = PC3), covering the 45.83% of the loadings. Linear discriminant analysis (LDA) and spatial distribution of **(B)** the Canonical Variables 1 and 2 and **(C)** box plot of values of the Canonical Variable 1 with the statistical groups including CTRL (*n* = 33), PD (*n* = 23), and AD (*n* = 10). ***p < 0.001, one-way ANOVA test. **(D)** Receiver operating characteristic (ROC) curve with the relative confidence interval (97–99%).

In order to verify if the observed differences could lead to the creation of a classification model able to discriminate the signals collected from CTRL, PD, and AD subjects, we performed the PCA–LDA on the collected spectra. The results are reported in Figure 4. The scatterplot of the first three loadings obtained by means of the PCA (cumulative PC scores = 46.2%; Figure 4A) shows a partial overlap of the data dispersion associated to the three defined groups represented on the base of the first three PCs with the higher loading (PC1 = 30.6%; PC2 = 8.9%; and PC3 = 6.5%, Figure 4A). The subsequent LDA on the first 10 PCs demonstrated a distinct dispersion of the Canonical Variables (CVs), with the CTRL, AD, and PD group means widely spaced (Figure 4B). Only a partial overlapping was observed between CTRL and PD data. Taking into consideration the dispersion of CV1, the differences between each group were proved to be statistically significant (*p* < 0.001, one-way ANOVA test, Figure 4C), indicating the potential role of RS in the discrimination of the salivary spectra acquired from PD respect to CTRL and AD.

The LDA analysis was used to perform a LOOCV and to create a classification model, using the acquired data for the training of the machine. The discriminatory performances of the model are reported in Table 3. The error rate for cross-validation of data was 1.05%, with calculated values of sensitivity, specificity, and accuracy of respectively, 97, 98, and 98%. The quality of the binary classification (PD versus no PD) was evaluated through the MCC with a related value of 0.97. The ROC curve, showed in Figure 4D, presents an area under the curve of 0.98 with standard error of 0.002 and confidence interval of 97% (significance level *p* < 0.001).

**TABLE 3:** Sensitivity, specificity, accuracy, Matthews correlation coefficient (MCC), error rate for the cross validation and area under the curve for the receiving operators characteristic (AUC ROC) curve for the single spectrum Parkinson’s disease leave-one-out cross-validation (PD-LOOCV) classification model.

	**Sensitivity**	**Specificity**	**Accuracy**	**MCC**	**Error rate**	**AUC ROC**
PD-LOOCV	97%	98%	98%	0.97	1.05%	0.98

#### Patient Level Classification Model

**FIGURE 5:**
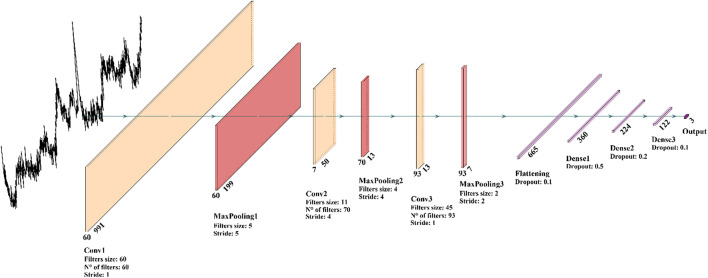
Graphic representation of the best 1D-CNN model configuration obtained through the hyper-parameters optimization process.

The application of DL techniques to spectral data involves a series of challenges. As result of the high complexity (and capacity) of such DL models, the available data volume should be large enough to create a more uniform and coherent dataset, and for this reason a data augmentation protocol was applied resulting in the generation of synthetic spectral examples to favor the network training and to boost the classification performances, allowing for better generalization capability. Furthermore, higher model complexity leads to a large number of possible configurations. Therefore, a suitable DL architecture must be selected by searching for the optimal composition of the various layers. Especially for CNNs, the configuration design requires a great effort in selecting the hyper-parameters. To this extent, we used SMBO to optimize the CNN architecture and fine-tune its hyper-parameters. Figure 5 shows the final configuration of the model.

**FIGURE 6:**
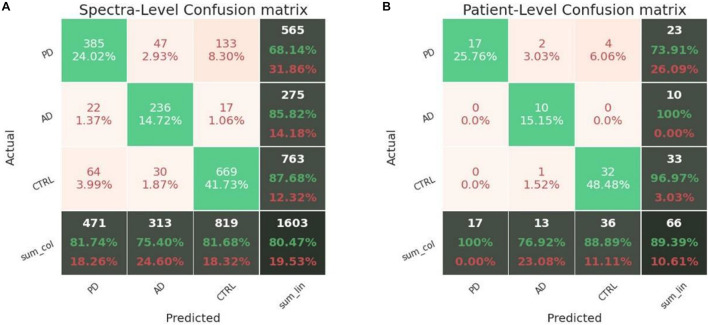
Confusion matrices obtained in LOPOCV by the proposed CNN. **(A)** Spectra-level and **(B)** patient-level.

Our CNN architecture consists of three 1D convolutional layers for the feature extraction and three fully connected layers for the classification. Comparing our DL model against the ML baseline introduced in section “Application of Learning Models for the Patient Classification Model,” we found that the ML models were systematically outperformed by the CNN-based model. This can be explained by the capability of a convolution-based model to capture the local structure in high dimensional complex Raman data, correctly elaborating the peak-related local correlations and information. Our findings about the suitability of DL models for Raman spectral analysis seems to be confirmed also by Liu et al. (2017) and Ho et al. (2019). Given the difficulty of the learning problem, characterized by a low-volume high-dimensional dataset, both the ML and DL models have been trained on pre-processed data. In addition, we performed classifications on raw unprocessed data, and we verified, in good agreement with Liu et al. (2017), that DL models, in particular CNNs, are capable of gaining competitive performances without the need of any preprocessing on the data. The DL architecture is trained on the three-class classification problem outputting a probability distribution associated with the class choices, where the highest probability is used to predict the label. The model has been tested using LOPOCV and performance breakdowns are reported in the confusion matrix in Figure 6. We firstly build a single-spectrum classification model (spectral-level) and then, having acquired multiple spectra for each patient, we aggregated the classification result at the patient-level according to their majority label. The average spectral-level sensitivity, specificity, and accuracy were respectively of 80, 89, and 80% using the CNNs (Table 4, spectral-level). In comparison, the more common ML techniques of SVM and RF achieved discriminatory power not comparable with the scores reached by the CNN.

**TABLE 4:** Accuracy, sensitivity, and specificity in patient- and spectra-level obtained by the proposed CNN in leave-one-patient-out cross-validation using a 10× data augmentation.

	**Sensitivity (%)**	**Specificity (%)**	**Accuracy (%)**
Spectral-level	80	89	80
Patient-level	90	94	89

The labeling decision for a patient can be taken based on its entire spectral set (since numerous spectral samples are acquired from the same individual). Classification metrics can thus be condensed into a new confusion matrix grouped by patients (Figure 6B), where the average sensitivity, specificity, and accuracy score of the CNN model were respectively of 90, 94, and 89%. Again, the scores of the tested ML methods were not comparable with the values reported for the CNN model.

#### Correlations

**FIGURE 7:**
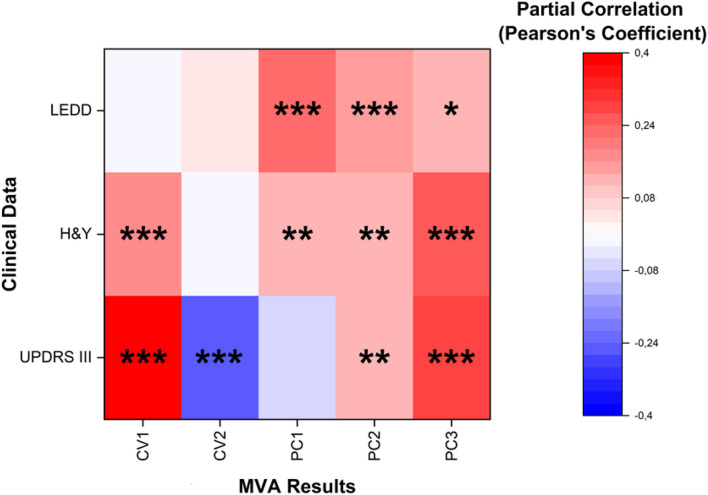
Heat map representing the partial correlation (Pearson’s coefficients) with the relative significance of Canonical Variables 1 and 2 (CV1 and CV2) and Principal Components 1, 2, and 3 (PC1, PC2, and PC3) correlated with levodopa equivalent daily doses (LEDD), Hoehn and Yahr (H&Y) stages and Unified Parkinson’s Disease Rating Scale (UPDRS) motor scales (III). Age, sex, and behavioral parameters were used as control covariates for the partial correlation. *p < 0.05, **p < 0.01, and ***p < 0.001, Pearson’s test.

Data extracted from the MVA of the Raman database were correlated with clinical and paraclinical parameters collected from the PD patients including UPDRS III, H&Y, and LEDD. In order to assess the independency of the Raman method, all the data were correlated using as correcting covariates the demographical data of the subjects, including age and sex. The results are reported in Figure 7. Interestingly, all the coefficients extracted from the Raman database using the MVA approach, show strong correlation with at least one of the indicated parameters. In particular, the clinical scales UPDRS III and H&Y demonstrated the influence on all the CVs and PCs correlated (Figure 7). The levels of levodopa influence mostly the PCs distributions, with strong positive correlation for PC1, 2, and 3, but not of the obtained CV1 and 2 (Figure 7) that represent the new set of coordinates in order to maximize the differences between the samples. A possible explanation for this result could be found in the influence of the drug therapy, which is able to influence the biochemical composition of saliva but not determining a direct influence on the final set of CVs used to build the classification model. The PCs represent independent directions, with their own specific weights (loadings, Figure 4A), applied in order to maximize the variance between the variables considered during the PCA. The data reported in Figure 7 indicate a strong dependency of the Raman data with the clinical status and stage of the PD patients.

## Discussion

The proposed pilot study paves the way to the possibility to use the entire salivary Raman spectrum of PD patients to assess the pathology onset and progression. The preliminary data presented here have proved that the Raman analysis of saliva can distinguish PD patients with sensitivity, specificity, and accuracy, respectively of 90, 94, and 89%. The importance of biomarkers in the great part of pathologies relies on the possibility to assess the disease onset, to shorten the diagnostic delay, to evaluate the disease progression and to perform a continuous monitoring of the efficacy of both therapeutic and rehabilitation strategies. Due to the various different forms of PD-related pathologies and to the overlapping of symptoms with other neurodegenerative diseases, the necessity to discover a specific biomarker, able to identify the early pathological onset, is of crucial importance. In the last decade, various studies proposed different potential biomarkers, collectable from peripheral blood or CSF with invasive procedures, but their potential application is still unclear (Parnetti et al., 2019). One of the reasons for the controversial results can be found in the pleiotropic role of the candidate molecules that can be associated to neurodegeneration in multiple neurological diseases, leading to a complex situation for the determination of a single biomarker uniquely associated to the PD onset and progression (Zhang et al., 2008). Previous studies have already demonstrated the applicability of the RS as diagnostic, prognostic, and therapeutics/rehabilitative monitoring tool using different biofluids for various neurodegenerative diseases (Devitt et al., 2018). In this work, we optimized a RS-based analysis for the spectroscopic characterization of saliva collected from PD patients. The optimized procedure allowed the collection of a highly informative signal from saliva, with information on proteins, lipids, carbohydrates, and nucleic acids, using a fast (20 min from the saliva collection to the Raman results), cheap (the only consumable reagent is commercially available aluminum), reproducible and minimally invasive procedure. Moreover, refining our previous sample preparation procedure and removing the filtration step (Carlomagno et al., 2020a), we were able to concomitantly amplify the range of analyzable molecules and increase the technique velocity. Concerning the economic burden of Raman spectroscopy use in clinics, we would like to mention that portable instruments are already commercially available on market, with different degrees of spectral resolution depending on the methodological needs and affordable for any diagnostic laboratory. We foresee that, once the methodology will be verified with the benchtop instrument in a larger cohort, the transferability of the method to a portable cost-effective platform will be evaluated.

By adopting the described technique, we were able to characterize the salivary Raman fingerprint of PD patients and to identify evident differences compared to CTRL and AD subjects, principally regarding the peaks and bands related to proteins, nucleic acids, saccharides, and lipids. In detail, the differences between PD and CTRL regard peaks related to proteins (829, 939, and 1001 cm^–1^ are signals from specific amino acids, while 1102 and 1346 cm^–1^ due to the Amide bands), nucleic acids (1244 cm^–1^) and glycoproteins/saccharides (850 and 1444 cm^–1^) (Movasaghi et al., 2007; Virkler and Lednev, 2010). The protein signal alterations can potentially derive from the pathological species present in saliva during the PD progression or due to the secondary effects caused by the pathological state (e.g., inflammatory responses or ROS damages). For example, high concentrations of α-synuclein, heme-oxygenase-1, protein damaged by ROS and the cysteine protease DJ-1 were found in the saliva of PD patients, which could explain the higher signals related to protein found in the subtraction spectra between CTRL and PD (Kang et al., 2014; Song et al., 2018; Bougea et al., 2019; Maciejczyk et al., 2020). Besides, the marked saccharides alteration can be related to the altered metabolism of glucose and related carbohydrates in PD patients, leading to an incremented amount of circulating saccharides (Van Woert and Mueller, 1971). Examining the spectral differences between PD and AD patients, those related to protein can be explained by the inflammatory species and aggregated proteins found in the circulating protein patterns in the two pathological states (Berg, 2008). On the other hand, the phosphatidylinositol accumulation in PD patients is probably due to the alteration of the phosphatidylinositol transfer protein expression responsible for the transport and metabolism of the phospholipid in PD (Chalimoniuk et al., 2006). The LDA model applied to the three experimental groups (PD, AD, and CTRL) revealed two CVs dispersed with a distinct trend, which allows to statistically discriminate the Raman single spectrum on the base of the CV. These results mean that the differences between the Raman spectra collected from the saliva of PD patients are significant enough to determine a classification model able to discriminate them from the CTRL or AD with accuracy, sensitivity, and specificity of more than 95%. The ML and DL approaches were refined and performed in order to automatize the signal preprocessing and, more importantly, to create a classification model able to discriminate not only the signal coming from a single spectrum, but also the entire Raman spectral set associated to the subject. This approach is one of the most advanced procedures for the creation of a fast and sensitive Raman-based diagnostic and monitoring tool close to the clinical application. The deep convolutional model that obtained the best performances has been trained on the pre-processed data. Nevertheless, competitive performances have been also reached by training the DL model directly on raw data, in good agreement with Liu et al. (2017). This can be explained by the fact that CNN models have local connectivity and translational invariance properties well suited for dealing with spectral data, where a vertical and horizontal translational invariance (e.g., small changes in intensity and in Raman shift) play important roles. These properties allowed a better handling of raw spectral data through CNNs, preventing the introduction of biases and information filtering during the manual data manipulation. The ML/DL pipeline resulted in the proper classification of almost all of the considered subjects. The attribution of part of the total number of PD spectra to the CTRL group (8.3% in the spectral-level confusion matrix) and consequently of four PD patients to the same group (patient-level confusion matrix), could be derived from the patients’ pathological progression. In fact, these particular patients had the lowest H&Y and UPDRS III clinical scores, determining the attribution by the classification models in the CTRL group, despite the regular assumption of the dopaminergic therapy. The results of this pilot study highlight the potentialities of the model not only to discriminate the pathological onset, but also to potentially identify the shades of the different PD stages. This feature must be validated with the study of new samples collected from a wider population of PD patients recruited at different pathological stages. Moreover, the introduction of new experimental groups and sub-groups related to the same pathology (e.g., different pathological stages and comorbidities), will be of fundamental importance to further train the classification mechanism, such as patients in the prodromal phase or parkinsonism followed in a longitudinal study and collecting the saliva samples at specific time-points corresponding to the pathological evolution. In the same way, data regarding the rehabilitation and drug therapies could provide an indication of the effectiveness of the prescribed procedures. In fact, one of the greatest advantages of this approach consist in the possibility to easily train the model with new and different data related to the target pathologies, leading to a refinement in the discriminatory power and to the classification of new groups, including PD stages of progression, overlapping comorbidities and PD prodromal phases. It has to be noted that the main limitations of the present work rely in the number of patients and in the involvement of a single laboratory for the RS analysis: as recently reviewed, multi-centered studies are needed to assess the actual robustness of RS methodology prior to their clinical application because of the profound influence of the setup-dependence in RS (Zech et al., 2018; Guo et al., 2020). For this reason, next steps in the validation of the proposed methodology will include the inclusion of larger cohorts of patients including different pathological stages and comorbidities, use of multiple instruments and involvement of different research teams to avoid experimental setup influence and biases in the validation phase.

Finally, our results are the first reported data that suggest the reliability of the Raman salivary analysis through the correlation of the MVA results with the clinical parameters of PD patients. In particular, despite the limited number of patients recruited, CV1 and CV2 demonstrated a statistically significant correlation (*p* < 0.001) with the two most relevant scales used nowadays for PD diagnosis and monitoring. The H&Y scale describes the motor symptoms correlated with PD progression, whereas the UPDRS provides a comprehensive tool to monitor PD related disability and impairment concerning a series of clinical manifestations including mental state, difficulty in performing daily activities, motor skills, dyskinesia, and others. Specifically, UPDRS part III refers to the motor function evaluation scale. The correlation of the CVs with UPDRS III and H&Y revealed a close relationship between the salivary biochemical content and the clinical state of the patient. It is worth noting that both scales have been recently reported to correlate also with the Raman biochemical fingerprint of serum extracellular vesicles in PD patients, assessing the close relationship between the biochemical equilibrium in biofluids and the disease onset (Gualerzi et al., 2019). This observation, together with the well-known physiological mechanisms of saliva production and the direct correlation of salivary composition to that of blood or CSF, make us hypothesize a possible involvement of the vesicular components in the salivary fingerprint of PD patients, although this issue requires further studies to be ascertained. Besides the partial correlation with the same clinical scales, the PC1, PC2, and PC3 also revealed a strong correlation with LEDD. These parameters could be exploited to individuate the optimal dose of dopaminergic therapy necessary to the specific PD patient, clinically framed by the associated Raman fingerprint. In order to investigate the molecular bases of these relationships and to validate the correlations between MVA data and clinical scales and therapies, further studies on larger and various cohort of PD are needed, including different PD progression stages and longitudinal studies.

Collectively, the MVA approach on the single Raman spectra allowed us to make a preliminary estimate of the diagnostic potential of the Raman analysis that was shown to reach an excellent accuracy level of 98.5%. Even more interestingly, the innovative ML/DL approach at the patient-level reached an accuracy of 89%. Once validated, this approach would represent a competitive diagnostic tool that could even surpasses previously proposed assays for PD (Saracchi et al., 2014). In conclusion, our pilot study demonstrated the potential application of the Raman analysis for the simultaneous identification of a large range of molecules present in saliva, obtaining high discrimination performances. The complex signal obtained from the salivary spectra was approached using a combined ML and DL method able to characterize and validate the Raman signature of PD and to assess with high discriminant ability the clinical state of the considered subject. Despite the small cohort of considered subjects, the potentialities of the proposed method were corroborated by the statistically significant correlation obtained between the MVA coefficients and the clinical data collected from the patients. The proposed methodology, once validated in larger cohorts and with multi-centered studies, could represent an innovative, cost-effective, minimally invasive, and accurate procedure to determine the early PD onset, progression and to monitor therapies and rehabilitation efficacy. Having in mind the future steps required for the final validation, we believe that this method has the potentiality to be transferred to the clinical setting, thus, the Raman analysis of saliva could provide clinicians and researchers with a powerful instrument for the PD managing.

## Data Availability Statement

The raw data supporting the conclusions of this article will be made available by the authors, without undue reservation.

## Ethics Statement

The studies involving human participants were reviewed and approved by institutional review board at IRCCS Fondazione Don Carlo Gnocchi ONLUS. The patients/participants provided their written informed consent to participate in this study.

## Author Contributions

CC, MB, AG, and SP conceived and designed the study. PB, MM, FV, NT, and VS collected the samples and clinical data. CC collected the Raman data. CC, SP, FR, and AG performed the statistical analysis. DB, MA, and EM created the classification model. CC, AG, MA, and DB wrote the original manuscript. All authors revised the manuscript and were involved in the drafting review, approving the decision to submit for publication.

## Conflict of Interest

VS received compensation for consulting services and/or speaking activities from AveXis, Cytokinetics, Italfarmaco, and Zambon. The remaining authors declare that the research was conducted in the absence of any commercial or financial relationships that could be construed as a potential conflict of interest.

## Publisher’s Note

All claims expressed in this article are solely those of the authors and do not necessarily represent those of their affiliated organizations, or those of the publisher, the editors and the reviewers. Any product that may be evaluated in this article, or claim that may be made by its manufacturer, is not guaranteed or endorsed by the publisher.

## Abbreviations

AD: Alzheimer’s disease
CNN: convolutional neural network
CSF: cerebrospinal fluid
CTRL: healthy controls
CV: Canonical Variable
DL: deep learning
FCNN: fully connected neural network
H&Y: Hoehn and Yahr
LDA: linear discriminant analysis
LEDD: levodopa equivalent daily doses
LOOCV: leave-one-out cross-validation
LOPOCV: leave-one-patient-out cross-validation
MCC: Matthews correlation coefficient
MCI: mild cognitive impairment
ML: machine learning
MVA: multivariate analysis
PC: Principal Component
PCA: principal component analysis
PD: Parkinson’s disease
ROC: receiver operator characteristic
RF: random forest
RS: Raman spectroscopy
SERS: surface enhanced Raman scattering
SMBO: sequential model-based optimization
SVM: support vector machine
TPE: Tree Parzen Estimator
UPDRS III: movement disorder society Unified Parkinson’s Disease Rating Scale motor part III.
