# Supplementary material for: Identification of the Raman Salivary Fingerprint of Parkinson’s Disease Through the Spectroscopic– Computational Combinatory Approach
Source: Front Neurosci. 2021 Oct 26;15:704963. doi: 10.3389/fnins.2021.704963 (PMC8576466; doi:10.3389/fnins.2021.704963)
Supplement: Supplementary file 1 [file Data_Sheet_1.pdf]

Supplementary Material

Identification of the Raman salivary fingerprint of Parkinson's disease through the spectroscopic-computational combinatory approach

Cristiano Carlomagno [1], Dario Bertazioli [2], Alice Gualerzi [1], Silvia Picciolini [1], Michele Andrico [2], Francesca Rodà [1], Mario Meloni [1], Paolo Innocente Banfi [1], Federico Verde [3,4], Nicola Ticozzi [3,4], Vincenzo Silani [3,4,5], Enza Messina [2], Marzia Bedoni [1]*.

[1] IRCCS Fondazione Don Carlo Gnocchi ONLUS, Milan, Italy
[2] Università degli Studi di Milano – Bicocca, Milan, Italy
[3] Department of Neurology – Stroke Unit and Laboratory of Neuroscience, IRCCS Istituto Auxologico Italiano, Milan, Italy
[4] Department of Pathophysiology and Transplantation, "Dino Ferrari" Center, Università degli Studi di Milano, Milan, Italy
[5] Aldo Ravelli Center for Neurotechnology and Experimental Brain Therapeutics, Università degli Studi di Milano, Milan, Italy

*** Correspondence:**
Corresponding Author: Marzia Bedoni, PhD. E-mail: mbedoni@dongnocchi.it

1 Details of the training phase and hyper-parameter values for the CNN model.

We performed an extensive computational analysis for comparing our DL model against ML baselines. We remark that, for a fair comparison, machine learning models have been trained after appropriate data preprocessing and model tuning (e.g. reducing the dimensionality via PCA, using preprocessed and normalized data, grid-searching the main hyperparameters).

We report quantitative results for the ML baseline in Suppl.Tab.1. To evaluate both ML and DL models at patient level, we applied a rigorous evaluation scheme, namely the Leave-One-Patient-Out CrossValidation (LOPOCV). We recall that Leave-One-Patient-Out Cross-Validation is a robust and stable procedure where each test-fold is composed of the entire set of spectra from a single patient (and the training-fold is composed of the spectra of the remaining patients). In limited data settings, like ours, this procedure gives an estimate of the model performance that is more accurate with respect to regular validation strategies (e.g. hold-out methods), avoiding possible classification biases given by the choice of an arbitrary set of patients' spectra as a test set.

The detailed results of the DL hyper parameter search, including the final values and the search spaces, are reported in Suppl.Tab.2. The CNN model is trained for a maximum of 200 epochs, using early stopping: therefore, for each fold of the LOPOCV, the training is stopped after 10 epochs of validation accuracy stagnation, to avoid overfitting. We exploited the *Adam* optimizer, scheduling its learning rate with a *ReduceLearningRateOnPlateau* strategy, starting from a learning rate value lr = 0.001 and systematically halving it once the validation metrics did not improve for 10 consecutive epochs. To boost the robustness and the generalization power of the data-hungry DL model, we generated new synthetic spectra by means of data augmentation procedure (Carlomagno, C. et al.,

2021). Indeed, it is possible to simulate the spectral imperfections and variations characteristics of the RS acquisition process by injecting a small contribution (equal ~0.1 σtrain, where σtrain is the intensity standard deviation of the training set) of Gaussian noise to the original spectra at random wavenumbers. In addition to direct noise injection, other augmentation components are applied, namely the stochastic modulation of the spectral offset and slope. The main effects include: our procedure slightly shifts the intensities, making the system invariant to weak translation along with the intensity axis; it reshapes the Raman peaks according to a certain multiplicative factor (1 ± 0.1 σtrain), and it alters the spectrum slope, from a local (close to a peak) or global perspective (slope was adjusted with a random multiplicative factor uniformly distributed in [0.95, 1.05]). In each augmentation step, the values of the additive/multiplicative parameters have been carefully chosen after an extensive exploration, having in mind that their variation range should be small enough to make sure that the generated spectra are still realistically similar to their original version, but, at the same time, large enough not to produce almost identical and therefore useless new examples. The data augmentation has been applied according to the optimized "data augmentation factor", but only to the training set, never to the test set. Since the performances have been measured in a LOPOCV, the data augmentation has been performed online. Notice that while applying LOPOCV, each training fold is augmented independently: in this way, we ensure no biases nor data leakage is introduced by the data augmentation procedure.

2 References

Carlomagno, C. *et al.* COVID-19 salivary Raman fingerprint: innovative approach for the detection of current and past SARS-CoV-2 infections. *Sci. Rep.* **11**, 4943 (2021).

3 Supplementary Tables

Model	Accuracy	Precision	Recall	F-Measure
**SVM**	0.851	0.825 (±0.12)	0.830 (±0.08)	0.821 (±0.08)
**RF**	0.836	0.824 (±0.06)	0.773 (±0.15)	0.791 (±0.08)
**FCNN**	0.851	0.834 (±0.04)	0.81 (±0.08)	0.82 (±0.04)

Supplementary Table 1. Accuracy, Precision, Recall and F-measure of the ML baseline models at the patients' level.

Hyper-parameters	Search Space	Final Value
**CNN1 filters**	int. [l = 5, h = 150]	60
**CNN1 filter size**	int. [l = 5, h = 100]	60
**CNN1 strides**	int. [l = 1, h = 5]	1
**MaxPool1 size**	int. [l = 2, h = 10]	5
**MaxPool1 strides**	int. [l = 2, h = 6]	5

**CNN2 filters**	int. [l = 5, h = 120]	70
**CNN2 filter size**	int. [l = 5, h = 30]	11
**CNN2 strides**	int. [l = 1, h = 5]	4
**MaxPool2 size**	int. [l = 2, h = 10]	4
**MaxPool2 strides**	int. [l = 2, h = 6]	4
**CNN3 filters**	int. [l = 5, h = 150]	93
**CNN3 filter size**	int. [l = 5, h = 20]	45
**CNN3 strides**	int. [l = 1, h = 5]	1
**MaxPool3 size**	int. [l = 1, h = 10]	2
**MaxPool3 strides**	int. [l = 1, h = 4]	2
**Dropout rate (after flattening)**	discrete unif. [l = 0.1, h = 0.95, q =0.05]	0.1
**Dense units 1 (first dense layer)**	int. [l = 32, h = 1024]	360
**Dropout1 rate**	discrete unif. [l = 0.1, h = 0.95, q =0.05]	0.5
**Dense units 2**	int. [l = 32, h = 1024]	224
**Dropout2 rate**	discrete unif. [l = 0.1, h = 0.95, q =0.05]	0.2
**Dense units 3**	int. [l = 32, h = 1024]	122
**Dropout3 rate**	discrete unif. [l = 0.1, h = 0.95, q =0.05]	0.1
**Batch size**	binned int. [l = 2, h = 512]	64
**Augmentation factor**	int. [l = 1, h = 100]	10

Supplementary Table 2. Summary of the optimized parameters for the final neural network hyper-parameters optimization phase with their relative search space and the final values obtained: int. refers to integer search space while logunif. and discrete unif. respectively to log-uniform and discrete uniform search space.
